# Unifying models of glioblastoma’s intratumoral heterogeneity

**DOI:** 10.1093/noajnl/vdaa096

**Published:** 2020-08-11

**Authors:** K H Brian Lam, Kristina Valkanas, Ugljesa Djuric, Phedias Diamandis

**Affiliations:** 1 Department of Laboratory Medicine and Pathobiology, University of Toronto, Toronto, Ontario, Canada; 2 Princess Margaret Cancer Centre, Toronto, Ontario, Canada; 3 Laboratory Medicine Program, University Health Network, Toronto, Ontario, Canada; 4 Department of Medical Biophysics, University of Toronto, Toronto, Ontario, Canada

**Keywords:** glioblastoma, glioma, heterogeneity, niches, single cell, therapeutics, transcriptomics

Glioblastoma (GBM) is an aggressive form of brain cancer with multiple layers of molecular and cellular heterogeneity implicated in treatment resistance and progression. Recent single-cell and regional transcriptional profiling efforts have highlighted a convergence of GBM’s molecular permutations onto a small handful of reoccurring cellular states and niche-specific programs. Understanding interdependencies between these intratumoral molecular phenotypes is critical to addressing treatment failures and could help further focus on the design of combination therapies for this deadly disease.

Glioblastoma (GBM) is the most common brain tumor type with a 15-month median survival despite multimodal therapy. Recently, molecular analyses have revealed that a key contributor to these treatment challenges lies in GBM’s heterogeneous biology. Large-scale genomic profiling initiatives^1,2^ highlighted that GBM consists of genetic subtypes that vary across patients. In adults, this primarily includes IDH-wildtype and mutated subgroups; the latter of which exhibit a more indolent clinical behavior.^1,2^ These interpatient differences have provided a framework for the development of therapies designed to target distinct biological drivers of GBM subtypes. In addition to these population-level variations, GBM also harbors intratumoral molecular heterogeneity that is differentially resistant to therapies and further challenges traditional treatment approaches.^3,4^ This phenomenon may explain why drugs designed to target the overall tumor biology, eventually fail due to the emergence of treatment-resistant clones. These intratumoral variations suggest that successful therapies will likely require the design of combination therapies where individual agents target different GBM subclones.

Many aspects of GBM’s intratumoral heterogeneity need to be considered before designing treatment strategies. Ideally, heterogeneity should be defined at the “phenotype” level by prioritizing transcriptional/proteomic readouts; molecules effectively targeted by current drug-based approaches. Similarly, while each patient has their own unique set of mutations, defining a finite set of recurring phenotypes across patients would allow for more generalizable “one-size-fits-all” treatments. Despite these intuitive prerequisites, only recently have tools become available to begin unraveling GBM’s intertumoral heterogeneity.

Using single-cell transcriptional profiling, GBM cancer cells were recently shown to exist in 1 of 4 distinct states.^5,6^ These states, defined by their transcriptional patterns, were termed astrocyte cell-like (AC-like), oligodendrocyte progenitor cell-like (OPC-like), neural progenitor cell-like (NPC-like), and mesenchymal-like (MES-like; Figure 1A). Importantly, these transcriptional patterns are plastic, with the ability of each cellular state to regenerate one another.^7^ The consistent presence of these 4 molecular phenotypes across patients provides a starting point for developing drug combinations that can simultaneously target and overcome heterogeneity-driven resistance.

**Figure 1.**
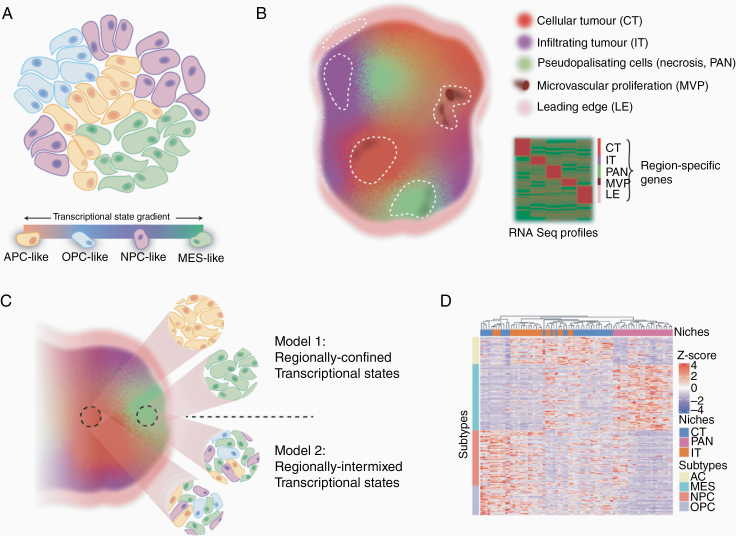
**Transcriptional models of intratumor GBM molecular heterogeneity.** (A) Single-cell transcriptomics capture GBM cells in one of at least 4 plastic transcriptional states which can evolve along a gradient over time. (B) Transcriptional profiling studies of laser-capture micro-dissected niches (dashed lines) highlight RNA signatures of hallmark histomorphologic regions of GBM. (C) Upper “interdependent” model of heterogeneity places the 4 plastic states positionally dependent on the microenvironment with some cell types being enriched in specific niches. The lower “nonoverlapping” model of heterogeneity proposes that the different GBM states are more randomly distributed across hallmark histologic features. If multiplicative, these different layers of molecular heterogeneity could effectively create many more GBM phenotypic subclones that could require individualized interventions. (D) Clustering the GBM cell state signatures across regional transcriptional profiles suggests regional enrichment of some cellular phenotypes.

Complementary approaches have resolved regional transcriptional profiles within GBM. Histologically, GBM cells show a relentless ability to infiltrate normal brain structures and form regions of severe hypoxia and necrosis, hallmark features implicated in relapse following surgery and chemoradiation therapy (Figure 1B). As such, other initiatives have focused on spatially isolating these regions using laser microdissection and exploring the specific molecular programs operational in their unique microenvironments.^8^ These approaches highlight consistent niche-specific molecular dependencies across GBM patients including hypoxia-inducible factor-related pathways in hypoxic niches, differentiation/proliferation programs in cellular regions, and tumor–brain interactions and stemness pathways in infiltrating zones. These regional analyses suggest that microenvironmental pressures may force GBM cells to adopt a small handful of reliable phenotypic molecular states that may also provide direction for combinatorial therapy design.

While these transcriptional approaches describe the existence of a relatively manageable number of GBM phenotypes, there is a need to begin integrating these layers of heterogeneity to discover areas of potential synergy or discord. For example, in one hypothetical model, the 4 defined transcriptional states of GBM cells could be interdependent and nonrandomly distributed throughout regional niches (Figure 1C). In this model, the overlap could provide cooperative programs that can be leveraged to address heterogeneity. Conversely, it is also possible that the 4 transcriptional states are stochastically found in each of these anatomical GBM niches. While this pattern could also suggest that addressing either one of these layers of heterogeneity may be sufficient to overcome resistance, it could also create more complex multiplicative variations that would not have been resolved by either study. If coexisting programs of cellular states and anatomical niches are in fact additive, it could result in as many as 12 coexisting states (eg, 4-distinct infiltrative, cellular, and hypoxic cell states). If each of these permutations has its own unique biology, it could make individual targeting of each subclone less feasible.

Integrating data from these landmark studies appears to preliminarily suggest a hybrid model.^6,8,9^ By examining the regional distribution of molecular signatures of each GBM cellular state across cellular, hypoxic, and infiltrating niches of GBM, a nonrandom, albeit imperfect, distribution is apparent, where transcriptional profiles of niches align with specific cellular identities (Figure 1D). For example, while the MES cellular state appears to align with hypoxic regions of GBM, the regional divide of AC, NPC, and OPC programs appears less certain. It is important to note that while such analyses are interesting, there are of course imperfect, as regional niches can be cellularly heterogeneous, especially at the infiltrating tumor border. Additional studies are therefore needed to understand the spatial single cell-level anatomy of GBM. In situ hybridization, immunofluorescence, or imaging mass cytometry-based approaches to examine coexpression of multiple markers corresponding to specific niches and cellular states could help better resolve which of these competing models of intratumor heterogeneity is most representative. It is also important to note that these models rely on transcript-level variation. As proteogenomic discordances^10^ become more apparent, understanding how cancer proteomes are spatially distributed across niches, and individual cells, could help further refine relevant GBM phenotypes requiring individual targeting.

Intratumoral heterogeneity is increasingly recognized as a major driver of failure in a number of clinical trials. While emerging tools allowed quantification of the intratumoral heterogeneity of GBM, it is important to understand the strengths and limitations of each approach. Integrating conclusions from emerging studies to build harmonious and unified models is essential. Here we present some possible multilayered models that integrate currently available information and provide direction and testable hypotheses that can be further explored to help better understand how intratumoral heterogeneity in GBM can be effectively managed.

## Notes

**Authorship statement.** All authors contributed equally.

## References

[1] Verhaak RG, Hoadley KA, Purdom E, et al. Integrated genomic analysis identifies clinically relevant subtypes of glioblastoma characterized by abnormalities in PDGFRA, IDH1, EGFR, and NF1. Cancer Cell. 2010;17(1):98–110. 20129251 10.1016/j.ccr.2009.12.020 PMC2818769

[2] Madhavan S, Zenklusen JC, Kotliarov Y, Sahni H, Fine HA, Buetow K Rembrandt: helping personalized medicine become a reality through integrative translational research. Mol Cancer Res. 2009;7(2): 157–167. 19208739 10.1158/1541-7786.MCR-08-0435 PMC2645472

[3] Bao S, Wu Q, McLendon RE, et al. Glioma stem cells promote radioresistance by preferential activation of the DNA damage response. Nature. 2006;444(7120):756–760. 17051156 10.1038/nature05236

[4] Calabrese C, Poppleton H, Kocak M, et al. A perivascular niche for brain tumor stem cells. Cancer Cell. 2007;11(1):69–82. 17222791 10.1016/j.ccr.2006.11.020

[5] Venteicher AS, Tirosh I, Hebert C, et al. Decoupling genetics, lineages, and microenvironment in IDH-mutant gliomas by single-cell RNA-seq. Science. 2017;355(6332). doi:10.1126/science.aai8478 PMC5519096 28360267

[6] Neftel C, Laffy J, Filbin MG, et al. An integrative model of cellular states, plasticity, and genetics for Glioblastoma. Cell. 2019;178(4):835–849.e21. 31327527 10.1016/j.cell.2019.06.024 PMC6703186

[7] Suvà ML, Rheinbay E, Gillespie SM, et al. Reconstructing and reprogramming the tumor-propagating potential of glioblastoma stem-like cells. Cell. 2014;157(3):580–594. 24726434 10.1016/j.cell.2014.02.030 PMC4004670

[8] Puchalski RB, Shah N, Miller J, et al. An anatomic transcriptional atlas of human glioblastoma. Science. 2018;360(6389): 660–663. 29748285 10.1126/science.aaf2666 PMC6414061

[9] Eberhart CG, Bar EE Spatial enrichment of cellular states in glioblastoma. Acta Neuropathol. 2020:1–3. doi:10.1007/s00401-020-02165-3 PMC7362280 32449056

[10] Djuric U, Lam KHB, Kao J, et al. Defining protein pattern differences among molecular subtypes of diffuse gliomas using mass spectrometry. Mol Cell Proteomics. 2019;18(10):2029–2043. 31353322 10.1074/mcp.RA119.001521 PMC6773564

